# Retraction: Secular Trends in Gastric and Esophageal Cancer Attributable to Dietary Carcinogens From 1990 to 2019 and Projections Until 2044 in China: Population-Based Study

**DOI:** 10.2196/101187

**Published:** 2026-06-16

**Authors:** JMIR Publications Editorial Office

**Affiliations:** 1 JMIR Publications Toronto, ON

The JMIR Publications Editorial Office is retracting the article “Secular Trends in Gastric and Esophageal Cancer Attributable to Dietary Carcinogens From 1990 to 2019 and Projections Until 2044 in China: Population-Based Study” [1].

This action is being taken due to identified concerns regarding the integrity of the peer-review process for this article. Following investigation, an author disclosed to the Editorial Office that they had impersonated an appointed reviewer for this article and fabricated a review.

Following this disclosure, the corresponding author Bing Hua Yin requested retraction of the article. This matter has since been referred to the institute for further investigation.

All of the authors agree with the decision to retract the article.
